# A synthetic high-voltage power line insulator images dataset

**DOI:** 10.1016/j.dib.2024.110688

**Published:** 2024-06-27

**Authors:** Reinaldo A.C. Bianchi, Hericles F. Ferraz, Rogério S. Gonçalves, Breno Moura, Daniel E.T. Sudbrack, Antoniele Merini, Maria de Lourdes G. Machado, Rodrigo Pires, Rafael Z. Homma

**Affiliations:** a Electrical Engineering Department, Centro Universitario FEI, São Bernardo do Campo 09850-901, São Paulo, Brazil; b School of Mechanical Engineering, Universidade Federal de Uberlândia, Uberlândia 38400-902, Minas Gerais, Brazil; c Centrais Elétricas de Santa Catarina, CELESC, Florianópolis 88034-900, Santa Catarina, Brazil

**Keywords:** Artificial intelligence, Machine learning, High voltage insulators, Object detection, Object classification

## Abstract

High-voltage power line insulators are crucial for safe and efficient electricity transmission. However, real-world image limitations, particularly regarding dirty insulator strings, delay the development of robust algorithms for insulator inspection. This dataset addresses this challenge by creating a novel synthetic high-voltage power line insulator image database.

The database was created using computer-aided design softwares and a game development engine. Publicly available CAD models of high-voltage towers with the most common insulator types (polymer, glass, and porcelain) were imported into the game engine. This virtual environment allowed for the generation of a diverse dataset by manipulating virtual cameras, simulating various lighting conditions, and incorporating different backgrounds such as mountains, forests, plantation, rivers, city and deserts.

The database comprises two main sets: The Image Segmentation Set, which includes 47,286 images categorized by insulator material (ceramic, polymeric, and glass) and landscape type (mountains, forests, plantation, rivers, city and deserts). Moreover, the Image Classification Set that contains 14,424 images simulating common insulator string contaminants: salt, soot, bird excrement, and clean insulators. Each contaminant category has 3,606 images divided into 1,202 images per insulator type.

This synthetic database offers a valuable resource for training and evaluating machine learning algorithms for high-voltage power line insulator inspection, ultimately contributing to enhanced power grid maintenance and reliability.

Specifications Table

**Table:** 

Subject	Electrical and Electronic Engineering.
Specific subject area	Power Transmission Lines maintenance using machine learning*.*
Type of data	Image.
Data collection	The database was created using computer-aided design software (Inventor and SolidWorks) and a game development engine (Unity 3D).
Data source location	The dataset was created at the Universidade Federal de Uberlândia, in Uberlândia, Minas Gerais, Brazil.
Data accessibility	Repository name: Zenodo Data identification number: 10.5281/zenodo.11287111 Direct URL to data: https://doi.org/10.5281/zenodo.11287111
Related research article	Hericles Ferraz, Rogério Gonçalves, Breno Moura, Daniel Sudbrack, Paulo Trautmann, Bruno Clasen, Rafael Homma and Reinaldo A. C. Bianchi. Automated classification of electrical network high‐voltage tower insulator cleanliness using deep neural networks. Accepted at: International Journal of Intelligent Robotics and Applications, 2024. https://doi.org/10.1007/s41315-024-00,349-8*.*

## 1 Value of the Data

• The dataset is valuable because even though it is synthetic, it accurately simulates real-world conditions with dirt and contaminants on insulators. This enables researchers to train and test algorithms without the need for a large collection of real-world images, which can be difficult or expensive to obtain.
• This dataset was created to allow researchers to train and test Computer Vision, Artificial Intelligence and Machine Learning algorithms without the need for a large collection of real-world images, which can be difficult or expensive to obtain.
• This dataset can be used to further develop and test AI algorithms. It can be used to train and test various AI algorithms for insulator inspection. Researchers can compare the performance of the proposed algorithms with their own or modify them for specific purposes. Also, the dataset can be used for benchmarking.

## 2 Background

Insulators are essential in high-voltage electrical systems, serving as a vital barrier between electrical conductors and the ground, preventing the flow of electrical current into the environment [1].

Nevertheless, insulators are not immune to damage and degradation. Regular maintenance and inspections are essential to ensure that insulators continue to function correctly. However, performing such maintenance tasks can be time-consuming, labour-intensive, and potentially dangerous for the workers involved. One solution to this problem is using autonomous systems, such as UAVs [2] and Robots [[3], [4], [5]], for preventive insulator maintenance.

Artificial intelligence techniques for maintenance have become widespread [6,7]. Machine learning algorithms require a large amount of data for training: models have a large number of parameters that need to be adjusted to learn patterns in the data. Additionally, considerable data is needed to avoid overfitting and account for outliers and data variation.

Unfortunately, real data on insulator strings are limited, especially in the case of dirty insulators. A real-world dataset must be collected, corrected, annotated, and checked. These steps need costly and time-consuming human labour.

To address this problem, we constructed a synthetic insulator image database [8] to develop, train, and test machine learning algorithms for this task [9].

## 3 Data Description

There are two main files on the dataset, containing two different set of images.

The first file (insulator-segmentation.zip) contains two folders: The folder "images" contains the images for insulator segmentation and is organized based on the insulator material type and the landscape in which the dataset was constructed. The folder "mask" contains the segmentation mask for the images.

The second file (insulator-classification.zip) contains images for insulator dirt classification and is organized based on the insulator material type and dirt type. It contains one folder called images. Inside it are 4 subfolders for each type of dirt: Salt, soot, excrement, and clean. This last one is for images without dirt.

Below is the folder structure for both files:



## 4 Experimental Design, Materials and Methods

**Fig. 1:**
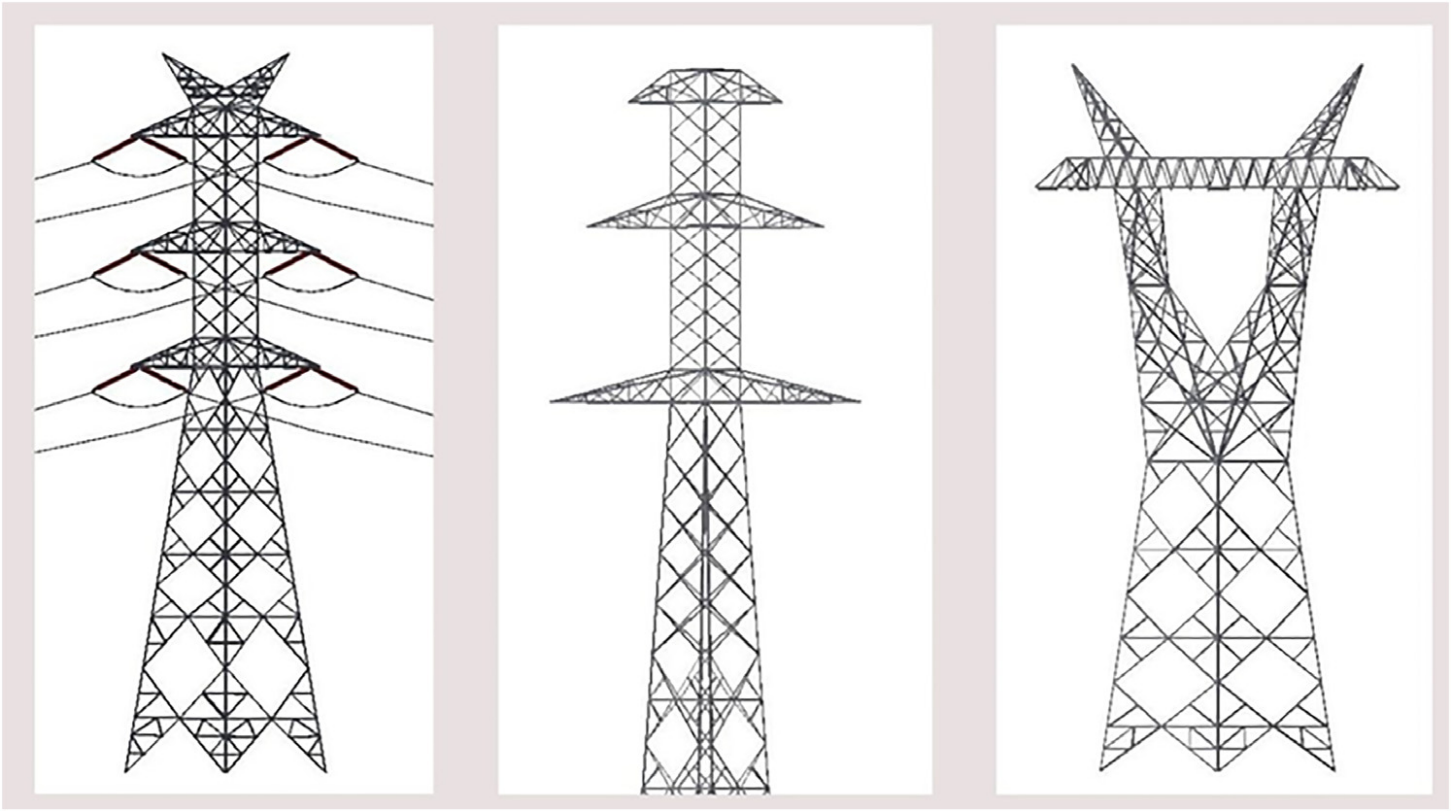
High-voltage transmission towers in the CAD software.

**Fig. 2:**
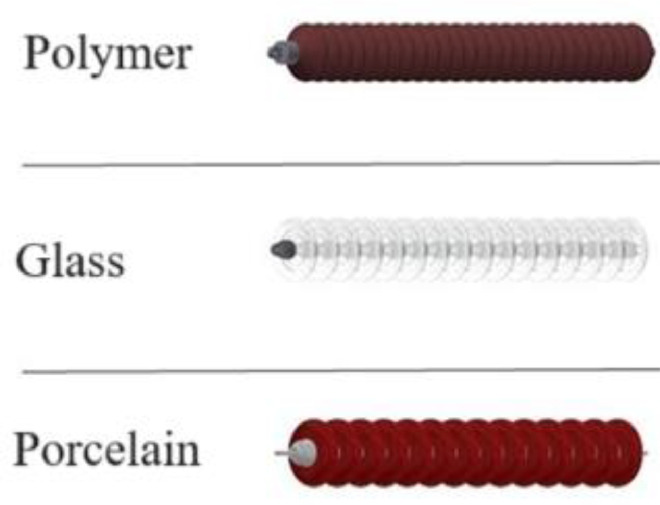
String insulators in the CAD software.

The first step in developing the dataset was importing publicly available towers and insulator models from CAD files into the CAD software used, in this case, Autodesk Inventor [10] and Dassault Systèmes SolidWorks [11]. In this stage, virtual tower assemblies were generated by combining the models of high-voltage electrical towers with the models of polymer, glass, and porcelain insulators, the most common categories used by energy companies. Fig. 1, Fig. 2 show High-voltage transmission tower and insulator models in Inventor software.

The next step in developing the dataset was importing the tower models into the game development engine used to model the virtual environment in which the images were made.

The game development engine used for developing the synthetic image database was Unity 3D [12], version 2020.3.32f1. Unity is a game development tool that supports the development of 2D and 3D games for multiple platforms. Unity 3D software offers several advantages for world and scene modelling, such as built-in features for physics simulation, lighting, and animation; real-time rendering; and an extensive library of pre-made 3D models.

Using Unity, several virtual environments were created using different landscape types, such as mountains, forests, deserts, cities, rivers, and plantations, where the towers were inserted. Using Unity lightning capabilities, different lighting conditions were simulated. Finally, several images in different positions and rotations were acquired for each environment using the camera tool. To capture images from different perspectives, the virtual camera was programmed to rotate at different angles around the axes that define the object's position, in this case, the string insulators 3D model, and to periodically move away from the focal point to reproduce different arrangements.

**Fig. 3:**
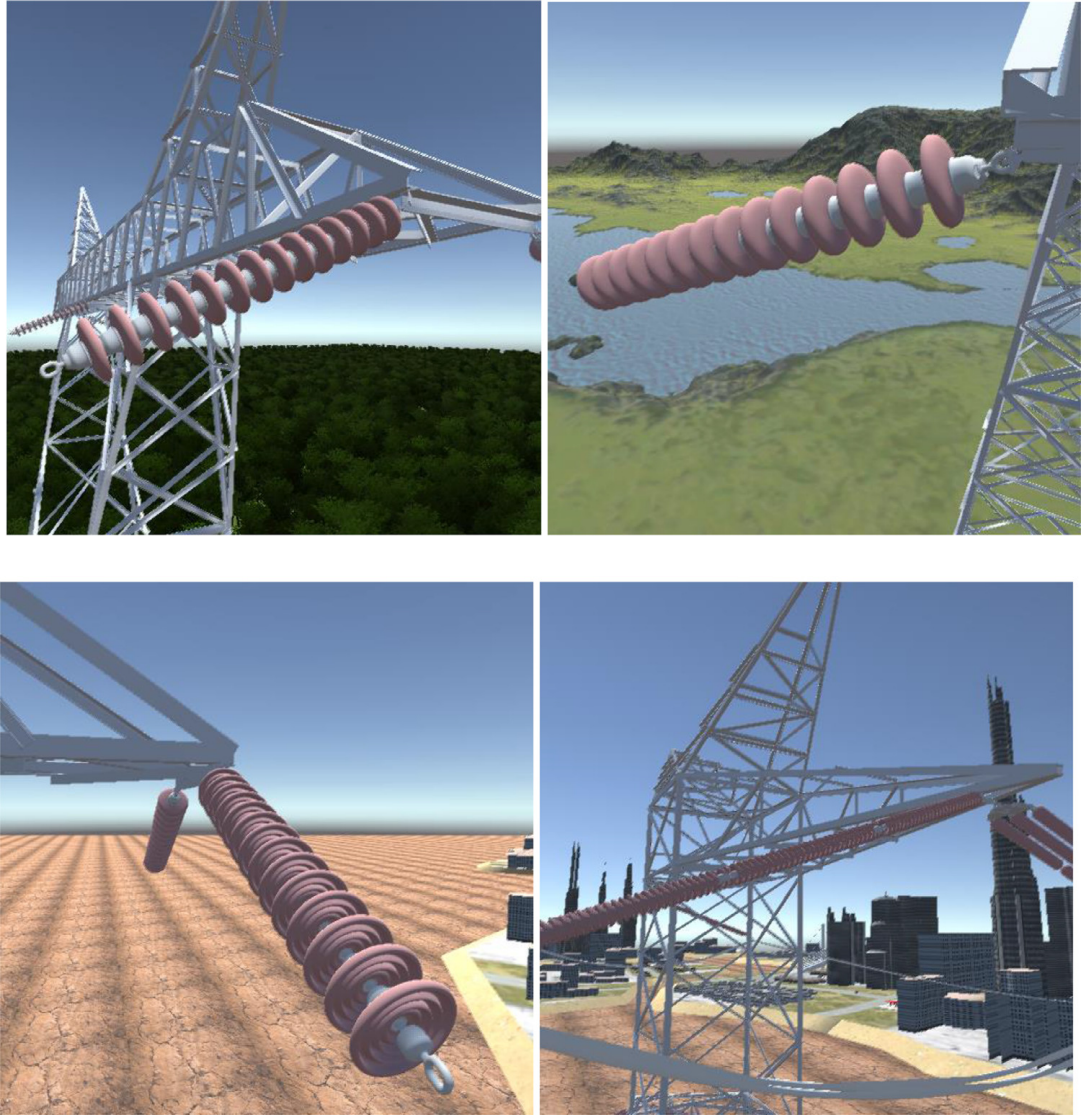
Samples of images in the dataset.

For each of the above-mentioned landscape types, 2627 images per disc type were created. Since the dataset considers a total of three insulator types, a total of 47,286 distinct images were created. Fig. 3 presents samples of these images.

**Fig. 4:**
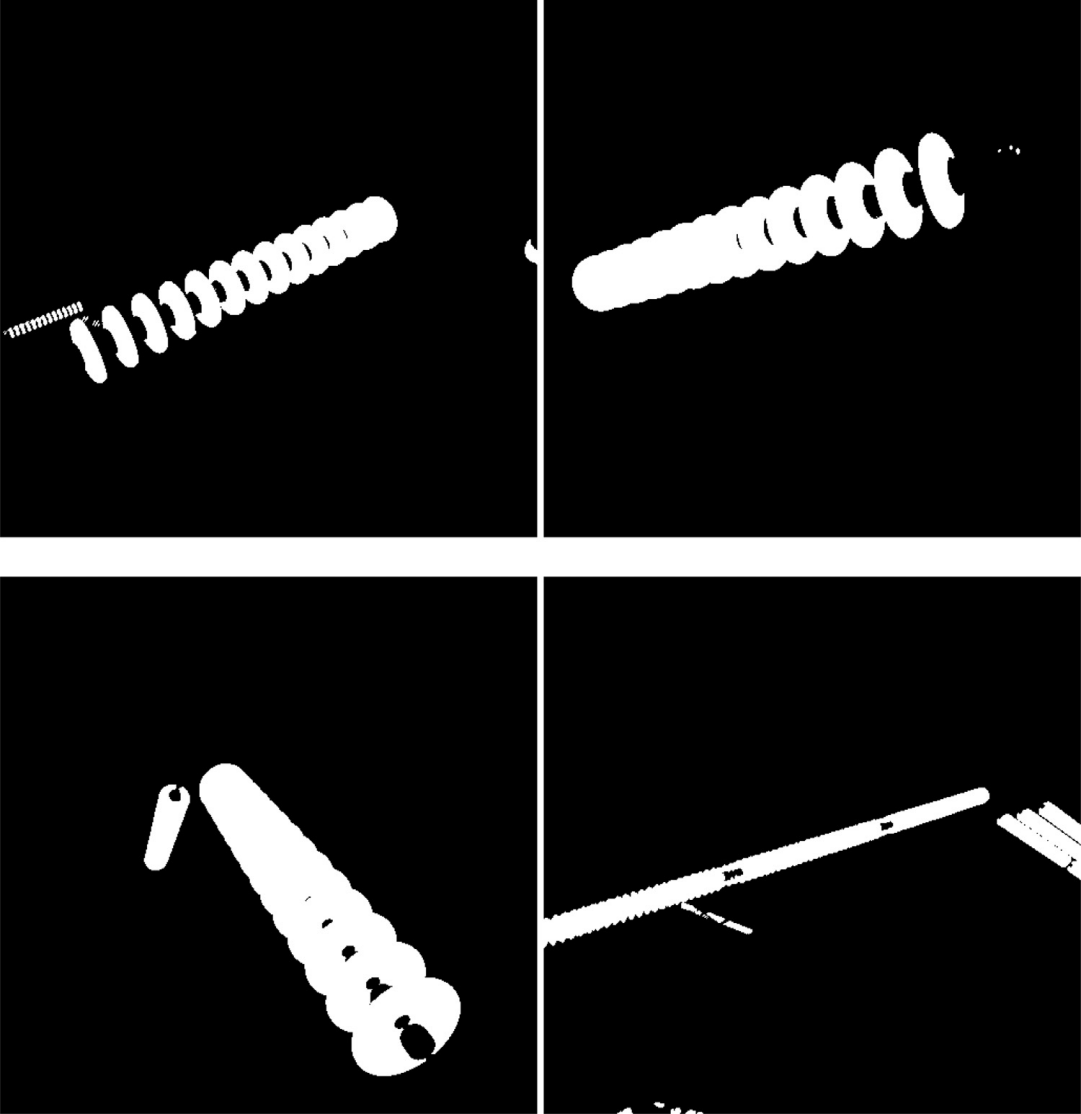
Samples of masks for the images in Fig. 3.

One advantage of the proposed methodology is the possibility of the automatic generation of the ground truth, called masks, that marks the position of the insulator in the images and are used in machine learning. To create the masks, the insulator colour in the images is changed to red, and a binarization algorithm was applied, where all red pixels are converted to white, and non-red pixels to black thus generating an image that represents the annotation of that particular image, as shown in Fig 4.

The second stage in creating this dataset was the development of images with dirt on the insulators, which were used for dirt classification. These images were constructed using Autodesk Inventor to simulate the most common impurities on high-voltage transmission line insulator strings. Three types of dirt were added to the insulator CAD images: soot, salt, and excrement. Textures representing each one were developed using Inkscape [13], a vector graphics editor, and then applied to the 3D models of the insulators in Inventor. Finally, images were generated using the same method of camera movements as in the first stage, creating the images for segmentation. The main difference between the two sets of files, apart from the dirt, is that for the classification images, no landscapes were used; only a standard blue colour was used as the background.

**Fig. 5:**
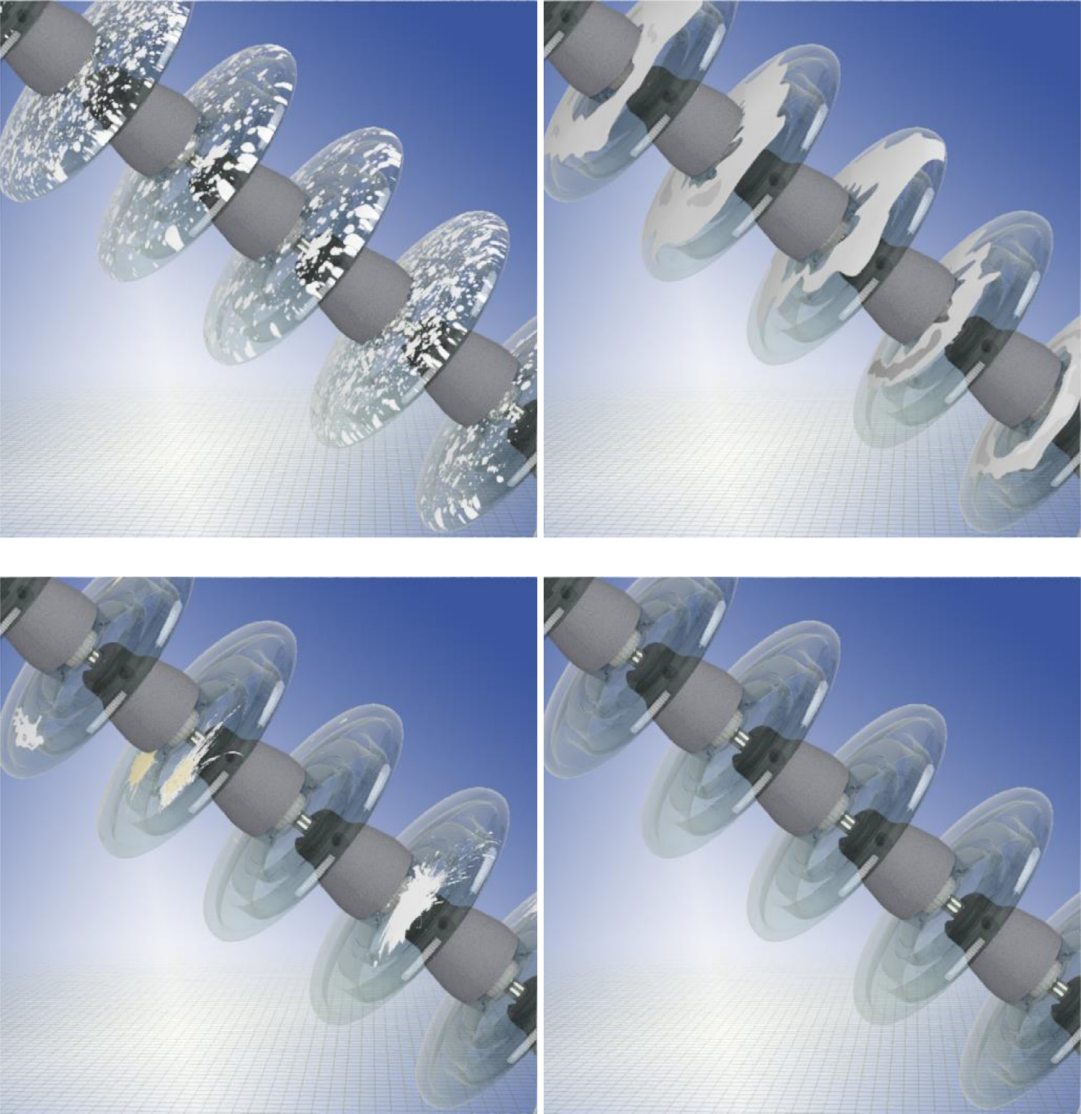
Images with dirt applied to the same glass insulator: salt, soot, bird droppings and the clean insulator.

Considering the four distinct types of impurities, including the clean insulator, the dataset contains is a total of 14,424 images with dirt. Samples of images with dirt are presented in Fig. 5.

## Limitations

Not applicable.

## Ethics Statement

The authors have read and follow the ethical requirements for publication in Data in Brief and confirm that the current work does not involve human subjects, animal experiments, or any data collected from social media platforms*.*

## CRediT authorship contribution statement

**Reinaldo A.C. Bianchi:** Writing – original draft, Writing – review & editing, Data curation. **Hericles F. Ferraz:** Software, Investigation, Validation. **Rogério S. Gonçalves:** Writing – review & editing, Conceptualization, Methodology, Supervision, Project administration. **Breno Moura:** Software, Investigation, Validation. **Daniel E.T. Sudbrack:** Writing – review & editing, Conceptualization, Funding acquisition. **Antoniele Merini:** Writing – review & editing, Conceptualization, Funding acquisition. **Maria de Lourdes G. Machado:** Writing – review & editing, Conceptualization, Funding acquisition. **Rodrigo Pires:** Writing – review & editing, Conceptualization, Funding acquisition. **Rafael Z. Homma:** Writing – review & editing, Conceptualization, Funding acquisition.

## Data Availability

Synthetic High-Voltage Power Line Insulator Images (Original data) (Zenodo).
